# Cancer 3D Models: Essential Tools for Understanding and Overcoming Drug Resistance

**DOI:** 10.32604/or.2025.067126

**Published:** 2025-09-26

**Authors:** Sofija Jovanović Stojanov, Marija Grozdanić, Mila Ljujić, Sandra Dragičević, Miodrag Dragoj, Jelena Dinić

**Affiliations:** 1 Institute for Biological Research “Siniša Stanković”-National Institute of the Republic of Serbia, University of Belgrade, Bulevar Despota Stefana 142, Belgrade, 11108, Serbia; 2 Institute of Molecular Genetics and Genetic Engineering (IMGGE), University of Belgrade, Vojvode Stepe 444a, Belgrade, 11042, Serbia

**Keywords:** Cancer three-dimensional (3D) models, cancer drug resistance, preclinical cancer models

## Abstract

Anticancer drug resistance remains a major challenge in cancer treatment hindering the efficacy of chemotherapy and targeted therapies. Conventional two-dimensional (2D) cell cultures cannot replicate the complexity of the *in vivo* tumor microenvironment (TME), limiting their utility for drug resistance research. Therefore, three-dimensional (3D) tumor models have proven to be a promising alternative for investigating chemoresistance mechanisms. In this review, various cancer 3D models, including spheroids, organoids, scaffold-based models, and bioprinted models, are comprehensively evaluated with a focus on their application in drug resistance studies. We discuss the materials, properties, and advantages of each model, highlighting their ability to better mimic the TME and represent complex mechanisms of drug resistance such as epithelial-mesenchymal transition (EMT), drug efflux, and tumor-stroma interactions. Furthermore, we investigate the limitations of these models, including scalability, reproducibility and technical challenges, as well as their potential therapeutic impact on personalized medicine. Through a thorough comparison of model performance, we provide insights into the strengths and weaknesses of each approach and offer guidance for model selection based on specific research needs.

## 1 Introduction

Cancer remains one of the leading causes of death worldwide, and a major obstacle to effective treatment is the ability of tumor cells to develop resistance to chemotherapy [1]. Despite advances in targeted therapies and immunotherapies, drug resistance continues to account for the majority of treatment failures and cancer-related mortality, necessitating new strategies to overcome resistance mechanisms [2,3]. Drug resistance in cancer includes both intrinsic resistance, where tumor cells are inherently non-responsive to therapy, and acquired resistance, which develops over time due to treatment-induced adaptations. Drug resistance in cancer is influenced by genetic mutations, epigenetic alterations, changes in drug efflux mechanisms [2,4]. In addition, it is reinforced by complex interactions within the tumor microenvironment (TME) [2,4]. Conventional *in vitro* models, especially two-dimensional (2D) monolayer cell cultures, have played a fundamental role in cancer research and drug screening. However, as they are unable to fully replicate the structural and functional complexity of *in vivo* tumors, their utility in studying resistance mechanisms is limited [5]. Two-dimensional cell cultures provide a controlled and reproducible environment for drug testing. However, they lack important features of solid tumors such as spatial heterogeneity and hypoxia gradients [6]. In addition, they do not replicate interactions with the extracellular matrix (ECM) or support three-dimensional (3D) cell-cell communication [6]. These factors are critical for drug response and resistance, and their absence in 2D systems often leads to discrepancies between *in vitro* and *in vivo* drug efficacy. While cancer cells in 2D cultures are uniformly treated with drugs, cells in tumors located in hypoxic or nutrient-depleted regions have altered metabolic status and increased resistance to therapy [7]. This fundamental difference has led to more and more studies using 3D models to close this gap and gain more clinically relevant insights. Because of their ability to mimic the structural and functional features of real tumors, 3D models offer a suitable platform for investigating both intrinsic and acquired resistance under conditions that reflect tumor heterogeneity, therapeutic pressure, and microenvironmental influences.

Three-dimensional tumor models have emerged as a more physiologically relevant alternative, bridging the gap between traditional monolayer cultures and *in vivo* tumors. These models better represent the architecture, heterogeneity and microenvironment of solid tumors, allowing for a more accurate representation of drug response [5]. However, different types of 3D models are suitable for studying different mechanisms of drug resistance, and the selection of the appropriate 3D model is crucial for studying the specific processes that contribute to resistance. For instance, spheroid models, which consist of self-assembled tumor cell aggregates, can effectively reproduce oxygen and nutrient gradients and have been widely used to study hypoxia-induced resistance and metabolic adaptations in a variety of solid tumor types, including breast [8], lung [9], ovarian [8], bladder [10], prostate [11] and brain tumors [9]. However, the extent to which these features are recapitulated may vary depending on tumor biology and growth characteristics. Organoid models derived from patients’ tumor tissue retain the genetic and epigenetic characteristics of primary tumors, making them an increasingly utilized tool for personalized drug resistance studies and precision oncology [12–14]. Hydrogel-based and ECM-embedded models mimic the mechanical and biochemical properties of tumor stroma, allowing researchers to study resistance mechanisms mediated by stromal interactions and ECM remodeling [15–17]. Alternatively, microfluidic “tumor-on-a-chip” systems incorporate dynamic fluid flow and endothelial barriers and are therefore suitable for studying drug transport, immune cell infiltration and resistance mechanisms associated with intratumoral drug penetration [18–20].

This review provides a comprehensive overview of the latest advances in drug resistance studies using 3D models. We analyze key findings from the recent literature that have expanded our understanding of how 3D tumor models can better predict response to therapies compared to traditional 2D cultures. We explore how different 3D tumor models capture different aspects of drug resistance and compare their strengths and limitations. Finally, we discuss how state-of-the-art 3D tumor models, characterized in recent research, can improve the predictive accuracy of cancer therapies in preclinical testing, ultimately leading to more effective cancer treatments.

## 2 Why 3D Tumor Models Are Essential for Translating Drug Resistance Research into Clinical Applications

A major challenge in cancer treatment is the failure of drugs in human trials, despite promising preclinical results. This failure is often due to the lack of predictive accuracy of 2D models. However, 3D tumor models offer a more realistic system that can better predict the behavior of tumors in patients [21]. By incorporating important aspects of tumor biology, such as cellular heterogeneity, drug penetration and the TME, 3D models allow for more reliable testing of drug efficacy and resistance [22]. This improved predictability makes 3D models a valuable tool for bridging the gap between laboratory research and clinical success.

One of the most promising applications of 3D tumor models is in the field of personalized medicine. Using models such as patient-derived xenografts (PDXs) and organoids, researchers can grow tumor cultures directly from patient biopsies [23]. These models preserve the genetic and phenotypic characteristics of the patient’s cancer, enabling specificity in drug testing and drug resistance studies. Three-dimensional models derived from patients reflect the full genetic diversity of the primary tumor, which makes them invaluable for determining the most effective treatment regimens for each patient [24].

In the clinical setting, a combination of different drugs can be used in cancer treatment to overcome resistance mechanisms [25]. Three-dimensional tumor models allow the interactions between different drugs to be studied in a more realistic setting compared to 2D models. By simulating the tumor’s response to the combination of multiple drugs, researchers can better understand how different drugs interact in the TME. This can help identify synergistic combinations or uncover potential drug-drug interactions that could lead to improved therapeutic efficacy or reduced resistance [26]. These findings are crucial for the optimization of chemotherapy protocols and the development of more effective treatment strategies.

As clinical trials become increasingly expensive and time-consuming, 3D tumor models offer a scalable alternative to traditional preclinical models, enabling prioritization of drug candidates and accelerating drug development [22]. These models are particularly valuable when it comes to high-throughput screening, allowing researchers to quickly identify promising candidates with the greatest potential for success in clinical trials. In addition, they enable the investigation of resistance mechanisms at a more detailed level, allowing decisions to be made about the most likely clinical pathways to investigate [27]. Ultimately, the use of 3D tumor models in the early phase of drug screening can significantly reduce the time and cost of clinical trials.

Cancer evolves in response to therapeutic pressure, and conventional 2D cultures offer limited insight into tumor adaptation because they cannot reproduce *in vivo* dynamics. In contrast, 3D tumor models better represent the continuous evolution of cancer under treatment pressure and allow researchers to study the development of tumor resistance over time [28]. By providing a more physiologically relevant environment, 3D models enable longitudinal studies that track changes in tumor behavior, gene expression and the development of resistant subpopulations. This long-term perspective is important to understand how tumors evolve and to develop strategies to prevent or overcome acquired resistance during treatment.

While 3D models offer a powerful improvement over traditional 2D cultures, especially in mimicking the tumor microenvironment and cellular behavior, they are not without limitations. Many 3D platforms still fall short in capturing systemic processes such as innervation, hormonal regulation, immune surveillance, and whole-body drug metabolism [29,30]. Moreover, complex biological phenomena like nerve invasion, vascular perfusion dynamics, or organ-organ interactions are difficult to replicate outside an organism [31]. Therefore, it is essential to view 3D models not as replacements, but as complementary tools alongside 2D monolayers and animal models [30]. While 2D systems remain valuable for mechanistic insights, cost-effective high-throughput screening, and early-stage compound evaluation, animal models provide a necessary systemic context that accounts for immune responses, pharmacokinetics, and metastatic progression. By integrating data across these platforms, using 2D for screening, 3D for tumor-specific and microenvironmental modeling, and *in vivo* models for system-wide validation, researchers can form a more comprehensive translational pipeline. This layered approach enhances the reliability of preclinical findings and supports the development of more effective, resistance-targeted cancer therapies.

## 3 Mechanisms of Drug Resistance in 3D Tumor Models

**Figure 1:**
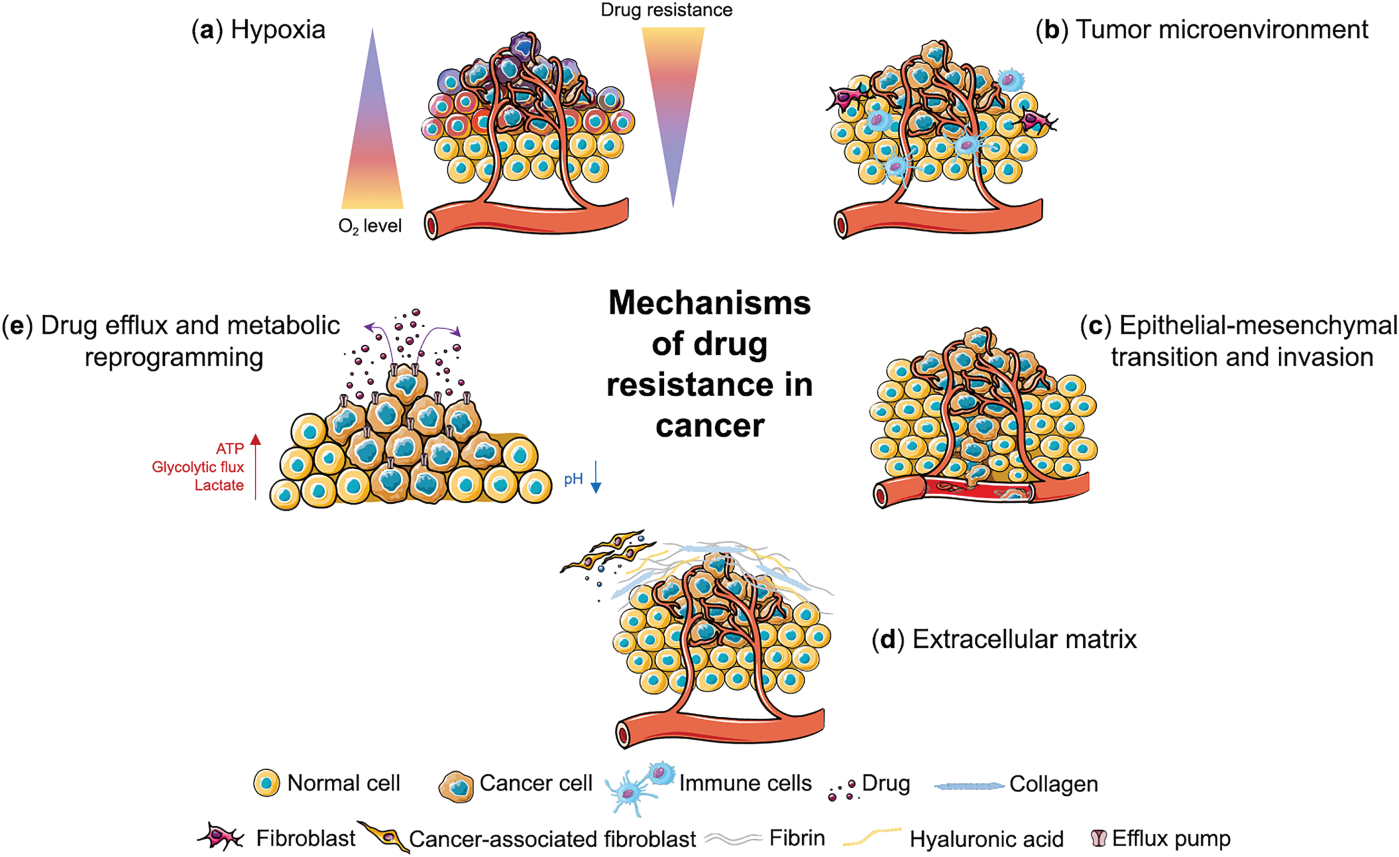
Mechanisms of drug resistance in cancer. Three-dimensional cancer models mimic different mechanisms of drug resistance including (**a**) hypoxia, (**b**) tumor microenvironment, (**c**) epithelial-mesenchymal transition and invasion, (**d**) extracellular matrix and (**e**) drug efflux and metabolic reprogramming. This figure was created using images adapted from Servier Medical Art (Servier, smart.servier.com, licensed under a Creative Commons Attribution 3.0 Unported License)

In 3D tumor models, cancer cells exhibit a variety of resistance mechanisms driven by the unique architecture and microenvironment of the tumor (Fig. 1). These mechanisms contribute to the ability of tumors to adapt to and survive therapeutic interventions. Several key factors are involved in the development of drug resistance, many of which are exacerbated or markedly different from those observed in 2D culture models. Below, we discuss the main mechanisms of drug resistance in 3D tumor models.

### 3.1 Hypoxia-Induced Resistance

Hypoxia is a hallmark of cancer and a critical feature of the TME, especially in the core of solid tumors [32,33]. Hypoxia occurs as the rapid tumor growth outpaces the development of blood vessels and the distance of cancer cells from functional blood vessels increases [34], leading to hypoxic regions with limited oxygen supply (Fig. 1a) (Table 1). Hypoxia has a major impact on the malignant phenotype of cancer cells by promoting proliferation, migration, invasion and angiogenesis [35]. It is associated with resistance to different types of therapies, tumor relapse and poor prognosis in various types of cancer [36–38].

**Table 1:** Investigating drug resistance mechanisms using three-dimensional (3D) models

Mechanisms of drug resistance	Overview	Suitable 3D models	References
**Hypoxia**	Tumor microenvironment with low oxygen	**Spheroids:** Commonly used to study hypoxia and tumor size/necrosis	[46–49,55–57,64]
	Promotes therapy-resistant cell populations	**Organoids:** Suitable for studying hypoxia-induced resistance, including angiogenesis and necrosis	
	Contributes to poor prognosis and recurrence	**Xenografts:** Suitable for studying the effects of hypoxia in an *in vivo* setting, but not ideal for precise control of hypoxic conditions	
		**Tumor-on-a-chip:** Can model hypoxia with microfluidics, allowing for control over oxygen gradients in the tumor microenvironment	
**Tumor heterogeneity**	Different cell populations within a tumor	**Spheroids:** Suitable for studying tumor heterogeneity and spatial drug exposure	[81,82,95,96, 98,100,119]
	Arises from genetic, epigenetic, and microenvironment changes	**Organoids:** Can replicate tumor heterogeneity and show metabolic gradients and cell-cell interactions	
		**Xenografts:** Useful for studying tumor heterogeneity *in vivo*, with spatial variation in drug exposure, but may not reflect the same diversity as *in vitro* models	
	Important in tumor progression and drug resistance	**3D bioprinted models:** Suitable for understanding heterogeneity in a more controlled 3D environment, with some limitations due to model size	
**Cancer stem cells** **(CSCs)**	Stem-like properties: self-renewal, differentiation	**Spheroids:** Commonly used to study CSCs due to their ability to form stem-like populations	[116–118,120]
		**Organoids:** Good for modeling CSCs and their role in resistance mechanisms	
	Found in regions with poor drug penetration	**Xenografts:** Can be used to study CSCs *in vivo*, particularly for personalized therapy approaches	
	Associated with therapy resistance and aggressive phenotypes	**3D bioprinted models**: Some suitability for studying stem-like properties, though less common for this purpose	
**Epithelial-mesenchymal transition (EMT)**	Increases cancer cell aggressiveness	**Spheroids:** Suitable for studying EMT because of their ability to form complex 3D structures, facilitating the study of invasive phenotypes	[121,122, 125,130]
	Makes cells more resistant to therapy	**Organoids:** Very suitable for studying EMT, especially for understanding transitions and metastasis	
		**Tumor-on-a-chip:** Useful for studying EMT in a controlled, dynamic environment where multiple factors can be modulated	
		**Xenografts:** Can be used to study EMT and its relation to tumor progression and therapy resistance *in vivo*	
		**3D bioprinted models:** Suitable for studying EMT due to their ability to manipulate ECM properties and cell organization, although less common	
**Cell-Extracellular matrix (ECM) interactions**	Cell-ECM interactions support tumor survival, ECM remodeling and secretion of factors that activate survival pathways	**Spheroids:** Useful for studying ECM stiffness, limited drug diffusion, and adhesion promoting EMT **Organoids:** Effective for studying ECM remodeling and interactions between cancer cells and stromal cells, such as cancer-associated fibroblasts (CAFs)	[77,136,139, 142,148]
		**Xenografts:** Suitable for studying ECM and CAF interactions *in vivo*, but may not capture all aspects of the microenvironment seen in 3D models	
		**3D bioprinted models:** Can be highly suitable for studying ECM stiffness, cell adhesion, and interactions with CAFs in a controlled environment, although less commonly used	
**Metabolic reprogramming**	Metabolic reprogramming (e.g., increased glycolysis) enhances cancer cell survival and resistance	**Spheroids:** Very suitable for studying metabolic shifts due to their 3D architecture, which mimics nutrient gradients found in tumors	[162–164,166,167]
		**Organoids:** Useful for studying metabolic reprogramming in a more realistic tumor microenvironment	
		**Tumor-on-a-chip:** Suitable for modeling metabolic reprogramming, especially in dynamic, controlled environment	
		**Xenografts:** Less suitable for studying metabolic reprogramming	
		**3D bioprinted models:** Suitable for studying metabolic reprogramming due to precise control over tissue architecture and gradients	
**Drug efflux pumps**	Drug efflux pumps reduce drug efficacy by pumping out therapeutic agents from cancer cells	**Spheroids:** Very useful for studying drug efflux, as they allow for real-time monitoring of drug penetration and resistance	[156–158,160]
		**Organoids:** Suitable for studying drug efflux in a more complex, organ-like environment	
		**Tumor-on-a-chip:** Useful for studying drug efflux in a microfluidic system with precise control over the environment	
		**Xenografts:** Could be used to study efflux pump activity *in vivo* but requires careful drug administration and monitoring	
		**3D bioprinted models:** Suitable for studying drug efflux dynamics by replicating tissue architecture and drug diffusion gradients	

Hypoxia is present in approximately 90% of solid tumors [32]. Tumors can contain regions with varying oxygen levels and with different types of hypoxia—chronic hypoxia, acute hypoxia, and cyclic hypoxia [39]. Chronic hypoxia is characterized by long-term exposure to hypoxic environment that persists for hours to days, and is present in cells that are distant from functional blood vessels. Acute or transient hypoxia occurs within minutes to hours due to transient disruption of blood flow, and cyclic hypoxia is caused by repeated cycles of hypoxia followed by reoxygenation.

Hypoxia-inducible factors (HIFs) are master regulators of cellular response to hypoxia eliciting downstream signaling that regulates angiogenesis, epithelial-mesenchymal transition (EMT), invasion/metastasis, cancer-specific metabolism, immune escape, and cancer stem cell maintenance [40,41]. HIFs are heterodimers comprised of an oxygen-regulated HIF-1α or HIF-2α subunit and a constitutively expressed HIF-1β subunit. Stabilization of HIF-1α and HIF-2α depends on the levels and duration of hypoxia, with downstream consequences dependent on the cellular and molecular context [42,43]. HIF-1 levels in tumor biopsy samples have been associated with high mortality rates and its targeting has been recognized as a promising route to improve therapy efficacy and reduce therapy resistance. Elevated HIF-1 levels and hypoxic signaling can be maintained even after cells leave hypoxic regions and re-enter the bloodstream, as demonstrated in circulating tumor cells in metastatic breast cancer [44] and more recently when transcriptional profile of these cells has been characterized [45]. A detailed understanding of signaling processes elicited by hypoxia are vital in finding new druggable targets in order to overcome hypoxia-induced therapy resistance.

3D cell models enable more relevance in studying tumor hypoxia than 2D cell models as they can mimic formation of hypoxic zones more accurately [46–48]. Oxygen levels during culturing have a profound effect on spheroid characteristics and have to be taken into account when designing a study. A recent study analyzing images of over 32,000 tumor spheroids identified key experimental parameters that influence the reliability of 3D tumor models, with oxygen levels being the most significant parameter affecting the spheroid size and necrosis [49]. This study showed that the oxygen levels under 3% were linked with reduced spheroid dimensions, a significant decrease in cell viability and increase in necrosis.

HIF-1α is the primary regulator of vascular endothelial growth factor (VEGF), a major regulatory cytokine that promotes blood vessel formation in solid tumors [50]. Although the use of 2D cell culture has been vital in revealing that HIF-1α is involved in VEGF expression [51,52], VEGF regulation in the 3D models mimics *in vivo* cases of tumor more closely and can provide insightful information on tumor angiogenesis and be used for screening drug candidates *in vitro*. Comparison of VEGF secretion between 2D culture of osteosarcoma and spheroid models under stress conditions showed increased secretion of VEGF in spheroids compared to cells in a monolayer [53]. Treatment with VEGF of 2D and 3D cultures of hepatoma cells showed that VEGF promoted tumorigenic properties in both models, however, invasion and angiogenesis were more aggressive in 3D cultures compared to 2D conditions [54].

Hypoxia is a major contributor to therapy resistance in cancer which has also been demonstrated by the use of 3D tumor models. Larger spheroids of triple negative breast cancer cells containing hypoxic core showed significant resistance to doxorubicin and cisplatin treatments as well as high expression of cancer stem cell marker cluster of differentiation 133 (CD133), compared to smaller spheroids without hypoxia [55]. Similarly, in colorectal cancer spheroids of different sizes representing normoxic, hypoxic, and hypoxic with necrotic stages, resistance to resistance to 5-fluorouracil was highest in spheroids including both hypoxic and necrotic areas [56]. A 3D tumor vasculature model of hepatocellular carcinoma (HCC) grown under hypoxic conditions exhibited increased resistance to axitinib compared to those under normoxic conditions [57].

Hypoxia promotes therapy resistance by different mechanisms, including impaired DNA damage response, altered cellular metabolism and promotion of therapy resistant cell populations. It disrupts DNA damage response in tumors, leading to DNA damage and increased replication stress, contributing to genomic instability [58–60]. Acute hypoxia leads to delay in DNA damage responses while chronic hypoxia leads to persistent suppression of DNA repair mechanisms promoting replication errors, double-strand breaks, and mutagenesis [61]. This dysregulation of DNA damage response limits the efficacy of therapy, and targeting checkpoint kinases such as ataxia telangiectasia and Rad3-related (ATR) protein, checkpoint kinase 1 (CHK1), WEE1 G2 checkpoint kinase (WEE1) and membrane-associated tyrosine- and threonine-specific cdc2-inhibitory kinase (MYT1) may improve treatment outcomes [62,63]. Use of 3D tumor models has enabled studying of DNA repair dynamics within 3D environments and evaluating effect of checkpoint kinase inhibitors in chemotherapy and radiotherapy [63–65].

Hypoxia, through HIF-1α, can also promote therapy resistance by increasing the expression of drug efflux pumps, such as P-glycoprotein (P-gp) that remove chemotherapeutic agents from cells, reducing their efficacy [66,67]. P-gp is a key mediator of chemotherapy resistance and expression of both HIF-1α and P-gp has been linked to increased resistance to chemotherapy in patients [68]. 5-fluorouracil (5-FU) treatment under hypoxic conditions was found to result in impaired apoptosis and increase in both P-gp and VEGF expression compared to normoxic conditions in gastric cancer cells [69]. Inhibiting P-gp, either directly or through development of HIF inhibitors, is an ongoing effort to reverse therapy resistance [68,70,71]. Comparisons between 2D and 3D cell models of different cancer cells have shown its increased presence in 3D models compared to 2D models, demonstrating higher innate resistance to anti-cancer drugs in 3D models and their potential in screening of novel drug candidates [72–74].

### 3.2 Tumor Heterogeneity and Spatial Drug Exposure

Tumor heterogeneity is another hallmark of cancer and refers to the existence of diverse cell populations with distinct genetic and phenotypic traits within a single tumor (intratumor heterogeneity) or between tumors of the same type in different patients (intertumor heterogeneity) (Fig. 1b) [75]. This structural complexity, which encompasses both tumor cells and the TME, is also dynamic as it evolves spatially and temporally during tumor development and leads to its constant reprogramming [76]. Tumor heterogeneity arises from multitude of factors—genetic mutations, epigenetic modifications, transcriptional and protein expression alterations, as well as from varying microenvironmental conditions within tumor and is considered to be a main contributor to the development of tumor progression and drug resistance [2]. Recent advancements in single-cell level high-throughput sequencing technology have provided an opportunity to explore the full complexity of the intratumor heterogeneity and hold great potential to identify biological markers and molecular targets in order to develop personalized treatment strategies [77–79].

Three-dimensional tumor models recapitulate the cellular heterogeneity of tumors and their dynamics more effectively than 2D models and enable the study of key features of solid tumors such as spatial drug exposure, metabolic gradients, and cell-cell interactions [80–82]. Their architecture resembles that of solid tumors, and depending on the size, with different cell layers including a necrotic core, an internal quiescent zone, an external proliferating region, and a gradient of nutrients and catabolites [83,84]. These models are therefore an invaluable tool in drug screening and preclinical studies, bridging the gap between 2D cell culture and animal studies [85]. Recent advancements in spatial transcriptomics have enabled precise mapping of gene expression in spatial context and better understanding of tumor heterogeneity within tissue architecture, demonstrating that the interplay between cellular plasticity and intratumoral heterogeneity is strongly dependent on spatial context [86–88]. Advanced imaging techniques, such as light sheet microscopy, enable visualisation of single cells even in a dense microenvironment of 3D tumor multiculture spheroids, enabling tracking of cell distribution and interactions in 3D environment during time and under the treatment [89,90].

In tumor tissue, drugs diffuse through layers of cells, forming concentration gradients that influence the response to treatment and its effectiveness [91]. Cells at the tumor periphery are exposed to higher concentrations of drugs, whereas those at the center of the tumor, in hypoxic or nutrient-deprived regions, are often shielded due to the presence of a dense ECM and limited vascularization. The limited ability of chemotherapeutics to reach all the cells in tumor tissue at high enough concentrations is the main obstacle to drug effectiveness. This property depends not only on the physicochemical properties of the drug and the protocols of administration [92], but also on the tumor vascularization, TME and ECM [93,94]. *In vivo* fluorescence lifetime imaging of tumor xenografts displaying differences in the amounts and distributions of TME components, such as collagen and vascularity, revealed a difference in distribution of trastuzumab, a monoclonal antibody that targets the human epidermal growth factor receptor (HER2), underlying the role of TME in drug availability within tumor [95]. Understanding the spatial dynamics of tumor heterogeneity and drug exposure is crucial in estimating drug effectiveness and state-of-the-art imaging techniques as well as spatial transcriptomic profiling, offer a valuable insight. Metabolomics profiling of PDXs of glioblastoma using 3D mass spectrometry imaging revealed distinctive spectral signatures from different regions of the tumor model, suggesting different metabolism and chemical environment of the cells in the core and the edge of the tumor [96]. This difference in the chemical environment could affect drug distribution by altering tissue drug affinity or transport, making it a critical factor to consider when developing therapeutic strategies. Three-dimensional matrix-assisted laser desorption/ionization (MALDI) imaging of paclitaxel distribution inside a 3D model of human malignant pleural mesothelioma revealed that the drug preferably localized in the edge of the tumors, at proliferating non-necrotic areas, while the lower drug signal corresponded to fibrotic and necrotic tumor core [97]. Similarly, MALDI imaging mass spectrometry of irinotecan distribution in HCT 116 colon carcinoma multicellular spheroids revealed time-dependent penetration of the drug into colon spheroids, with higher concentration of metabolites in the outer rim of the structure, suggesting a higher metabolism of the drug in viable cells at proliferative and quiescent zones of the 3D spheroids [98]. An approach utilizing integration of MALDI imaging, phosphoproteomics, and multiplexed tissue imaging for mapping of adavosertib, WEE1 inhibitor, distribution in PDX lines of glioblastoma, revealed that spatial distribution of the drug within tumors followed distinct histological features such as necrosis, DNA damage, tumor cell density, and hemorrhagic regions [99]. Advanced spheroid models, such as vascularized tumor spheroids, and use of light sheet microscopy, showed that distribution of cancer cells within TME and their interaction with it significantly varies between different cell lines [100]. It can present as a clear separation of tumor cells and stroma, a common histological feature in many cancers, or as a stochastic mix of cells where tumor cells are in direct contact with pseudovessels, corresponding to an invasive phenotype, which would therefore have different implications for drug distribution. Taking into account the differences in tumor composition and its influence on drug distribution holds a potential for improving precision medicine strategies in cancer treatment [101].

Spatial drug gradient leads to the selective survival of drug-resistant populations deeper within the tumor. Within these deeper regions, a minor subset of cells known as drug-tolerant persisters (DTPs) can avoid drug-induced cell death by adopting apoptosis-resistant state [102,103]. This initial resistance to therapy is carried out through metabolic adaptation and stress response mechanisms and does not involve genetic alterations [104]. Common mechanisms of avoiding drug-induced cell death include adopting features of the EMT [105], insulin-like growth factor 1 receptor (IGF-1R) signaling and an altered chromatin state as well as activating pro-survival cytokine signaling pathways such as interleukin-6 (IL-6) [106,107]. A signature apoptotic response of colorectal cancer has been recently characterized at single cell level helping identify drug tolerant persisters and guiding future therapies [108]. Once the selective pressure of the treatment is removed, DTPs may re-enter the cell cycle and contribute to relapse and metastasis, as they can evolve into more resistant clones over time [109]. DTPs appear at a very low frequency, depending on tumor type and drug treatment [104]. DTPs were first described in *in vitro* cell models [110] and their presence is now linked to long-term relapse in patients considered clinically disease-free and was found across a wide range of cancer types under diverse chemotherapies and targeted therapies. Novel therapies targeting DTPs are urgently needed and 3D tumor models offer invaluable tool for characterizing these cells in order to suggest more effective treatment options. Single cell sequencing of murine organotypic tumor spheroids undergoing programmed cell death protein 1 (PD-1) blockade, identified a discrete subpopulation of immunotherapy persister cells vulnerable to tumor necrosis factor alpha (TNF-α)–induced cytotoxicity [111]. Spheroids and patient-derived organoids (PDOs) of head and neck squamous cell carcinoma cell lines showed increased sensitivity to X-rays and proton beam therapy in the presence of either a CHK1 or a WEE1 inhibitor [63]. Protein kinase B (AKT)-driven proteasome targeting was found to reduce DTPs in breast, lung and ovarian cancer cells harboring phosphatase and tensin homolog (PTEN)/phosphoinositide 3-kinase (PI3K) pathway mutations [112]. A deeper understanding of the vulnerabilities of DTPs could enable strategies to target them early, potentially delaying or even preventing the development of acquired resistance, and further development of single-cell resolution technologies and 3D models will have a fundamental role in this.

Cancer stem cells (CSCs) are another cell population enriched in 3D models, often located in regions with poor drug penetration, such as hypoxic areas [113]. CSCs have stem-like properties such as the ability to self-renew, differentiate into diverse tumor cell types and to initiate and sustain tumor growth. They have prolonged life span compared to bulk of tumor cells, are frequently more resistant to conventional therapies, such as chemotherapy and radiation, and are associated with aggressive phenotypes of malignant tumors [114,115]. In 3D models, CSCs are better represented, and their survival in response to treatment is a key feature of the tumor’s overall resistance to therapy. Three-dimensional spheroids have been used to enrich CSCs in different types of cancer, including breast [116], brain [117], and head and neck cancer [118]. A recent paper describing vascularized tumor spheroids based on different breast cancer cell lines showed increased expression of cluster of differentiation 44 (CD44), marker of CSCs linked to chemoresistance, in vascularized tumor spheroids compared to 2D culture, except in cells already abundant in CD44 such as MDA-MB-435 cell line [100]. Characteristics of the spheroids in terms of enrichment with stem cell population differ depending on the method used as well as on the cell types. Significant improvement of the efficiency of cancer stem cell selection of breast cancer has been achieved using a low cell density on low-adhesion flat plates [119]. This paper also demonstrated that stem cells from patient-derived breast cancer tissue, selected and cultured in spheroids, were able to subsequently differentiate into breast cancer cells when transplanted into mice as xenografts. Circulating CSCs originating from pancreatic cancer were successfully used to generate tumorospheres and PDXs which then closely resembled the histological features of the primary tumor highlighting their potential in tailoring personalized therapy [120].

### 3.3 EMT and Invasiveness

Cancer cells undergoing EMT are often more aggressive and invasive, making them more likely to spread to other parts of the body (Fig. 1c). These cells are typically more resistant to therapy, including chemotherapy and targeted treatments. In 3D models, the induction of EMT can be more accurately modeled by replicating the TME and drug exposure conditions that promote this transition.

EMT is a central process in cancer in which epithelial cells lose their polarity and adhesion and acquire mesenchymal properties such as motility and invasiveness. EMT is responsible for tumor progression, metastasis and resistance to therapy. In contrast to 2D models, 3D tumor models—such as spheroids, organoids and microfluidic systems—can better replicate the complexity of EMT and its role in drug resistance by mimicking tumor architecture, hypoxic gradients and selective pressure. These models improve cell-ECM interactions and alter EMT-related gene expression and resistance mechanisms [121,122]. In non-small cell lung cancer (NSCLC) spheroids (e.g., A549 cell line), EMT markers (vimentin, fibronectin) and drug resistance proteins (glutathione S-transferase P1 (GSTP1), multidrug resistance-associated protein 1 (MRP1)) were upregulated in both scaffold-free and scaffold-based 3D models, correlating with increased migration and chemotherapy resistance [121]. Hypoxia in these models increased VEGF-A, which enhanced anti-apoptotic signaling and efflux pump activity, further promoting resistance [121]. This suggests that 3D cultures, especially scaffold-based spheroids, more accurately reflect tumor invasiveness and EMT dynamics than 2D systems.

EMT activates survival pathways such as Wnt/β-catenin, Notch and transforming growth factor beta (TGF-β), enabling resistance to therapy [123]. Mesenchymal-like cells often overexpress efflux pumps (e.g., P-gp), which reduces drug efficacy [124]. While 2D cultures oversimplify these mechanisms, 3D models provide a physiologically relevant context. EMT-driven resistance in 3D models is mediated by Notch, Wnt/β-catenin, and TGF-β signaling, which sustain CSCs, enhance chemoresistance, and promote invasion. In colon carcinoma spheroids, Notch activation increased stem cell markers (CD133, CD44) and mirrored patient-derived chemoresistant colon cancer cells, highlighting its role in the maintenance of CSCs on therapy [125]. ECM interactions, such as tenascin-C in breast cancer spheroids, enhanced stem cell formation via Notch signaling [126,127]. Nasopharyngeal carcinoma spheroids showed increased resistance to cisplatin, doxorubicin and paclitaxel, with RNA sequencing revealing upregulated Wnt/β-catenin signaling compared to 2D cultures [128]. In ovarian cancer spheroids, Wnt signaling drove macrophage activation and maintenance of CSCs and enhanced chemoresistance [129]. In ovarian cancer spheroids, TGF-β activated EMT genes by downregulating E-cadherin and upregulating N-cadherin, promoting invasion and resistance, responses that were less pronounced in 2D cultures [130]. Microfluidic models of lung cancer spheroids also showed that TGF-β regulates EMT under dynamic conditions [131].

EMT is also linked to an increase in stem cell-like characteristics in tumor cells. CSCs, characterized by their ability to self-renew and differentiate, exhibit increased resistance to therapy, making them an important target to overcome drug resistance in 3D tumor models [132]. EMT-associated acquisition of stem cell-like properties has been extensively studied across various 3D tumor models, including spheroids and organoids. In colorectal cancer spheroids, cyclic chemotherapy altered the expression of CSC markers, suggesting adaptive survival strategies [133]. Nasopharyngeal carcinoma spheroids showed higher stem cell marker expression and migration than 2D cultures [128,134]. CXCR4+CD133+ cells isolated from ovarian cancer cell lines exhibited high spheroid-forming capacity, increased resistance to therapy and high tumor formation capacity [135]. Breast cancer spheroids exhibited enriched CSC populations (CD44+/CD24-) after treatment with a HER2 inhibitor, reflecting therapy-induced stem cell formation [136]. PDOs were shown to retain CSC markers (aldehyde dehydrogenase (ALDH), CD133, CD44) and tumor heterogeneity, providing insights into stem cell-induced resistance [137,138]. A microfluidic platform study in pancreatic ductal adenocarcinoma linked the increase in CSCs to disease progression and underscored the role of EMT in tumor-initiating cells [139].

### 3.4 ECM and Cell-ECM Interactions

The ECM, composed of proteins such as collagen, fibronectin, and elastin, regulates the behavior and resistance of tumors (Fig. 1d). The ECM can act as a physical barrier that prevents drugs from effectively reaching tumor cells, particularly those located deep within the tumor mass. This barrier is especially important in solid tumors with dense stroma, where ECM components, including collagen and hyaluronic acid, can limit the diffusion of chemotherapeutic agents. 3D models represent the dynamics of the ECM better than 2D cultures and improve the understanding of resistance mechanisms. Various 3D culture approaches, including patient-derived scaffolds and spheroid models, have been used to study ECM-induced drug resistance. Breast epithelial organoids in collagen hydrogels showed that higher ECM stiffness increased invasion and resistance [140]. In breast cancer models using the MDA-MB-231 and MCF7 cell lines, patient-derived scaffolds enhanced CSC-like properties and EMT markers, suppressed differentiation and promoted resistance [141]. Moreover, MCF7 spheroids in collagen agarose matrices showed enhanced cell-matrix adhesion promoting EMT and resistance [142].

The ECM interacts with receptors such as integrins and CD44, activating survival pathways that lead to resistance [143]. These interactions can activate signaling pathways that protect cancer cells from the cytotoxic effects of treatment. In 3D models, these interactions are more pronounced, allowing for a better understanding of how the ECM contributes to drug resistance. For instance, in 3D cultures of HUVEC and MDA-MB-231 cells, blocking integrin αvβ3 reduced migration and tumor responses [144]. Lymphoma organoids with integrin-targeting peptides showed increased chemoresistance [145]. Hepatobiliary cancer organoids associated high CD44 expression with resistance via Janus kinase–signal transducer and activator of transcription (Jak-STAT) signaling [77]. In gastric cancer organoids, knockout of KHDRBS3 reduced CD44 variants and increased drug sensitivity [146].

In the TME, fibroblasts can differentiate into cancer-associated fibroblasts (CAFs), which promote ECM remodeling and tumor progression. CAFs can secrete factors such as TGF-β and IL-6 that promote cell survival and drug resistance [147]. In 3D models, the inclusion of CAFs enables a more realistic representation of the impact of stromal cells on drug resistance and treatment outcomes. In head and neck squamous cell carcinoma spheroids, CAFs increased tumor proliferation and EMT via epidermal growth factor receptor (EGFR) expression [148]. Prostate, pancreatic, breast and lung cancer spheroids showed CAF-driven growth [149,150]. In lung cancer spheroids, CAF-derived miR-196a was identified as an EMT regulator [151]. Liver tumor organoids with CAFs exhibited resistance to sorafenib and 5-FU [152]. In breast cancer spheroids, IL-6 derived from CAFs had an anti-apoptotic effect in contrast to 2D cultures [153]. CAFs can also recruit macrophages and, as a result, enhance resistance, as observed in NSCLC and breast cancer spheroids [154,155].

### 3.5 Altered Drug Efflux and Metabolism

Another important mechanism of drug resistance in 3D tumor models is altered drug metabolism and transport (Fig. 1e). In 2D models, cancer cells exhibit relatively uniform expression of drug transporters and metabolic enzymes, which often leads to misleading conclusions about drug efficacy. In contrast, 3D tumor models better represent the diversity of metabolic and transporter profiles present in different regions of the tumor.

Efflux pumps actively transport chemotherapeutics out of cancer cells, reducing intracellular drug concentration and efficacy. In 3D tumor models, key transporters such as P-gp, MRP1, and breast cancer resistance protein (BCRP) are frequently upregulated, contributing to chemoresistance [156]. This upregulation of efflux pumps is a key mechanism of resistance in the hypoxic and nutrient-deprived regions of the tumor. Studies using spheroid cultures have demonstrated BCRP’s role in glioblastoma self-renewal [157] and its contribution to spheroid formation and invasion in head and neck squamous cell carcinoma [158,159]. Furthermore, silencing ATP-binding cassette (ABC) transporters reduced CSC properties and EMT in NSCLC and breast cancer [160,161].

Cancer cells often undergo metabolic reprogramming to support their rapid growth and survival. In 3D cultures, this reprogramming is more pronounced, as tumor cells may shift to aerobic glycolysis or alter their mitochondrial function, which can contribute to resistance to treatments that target cellular metabolism or oxidative stress. Several studies have used 3D models to investigate metabolic reprogramming in cancer, highlighting the role of different metabolic pathways in resistance formation. Spheroids of colorectal and pancreatic cancer cells exhibited altered glucose metabolism compared to 2D cultures, with increased ATP production and metabolic shifts under nutrient stress [162]. In pancreatic ductal adenocarcinoma (PDAC), spheroids displayed a glycolytic shift and ECM remodeling, reducing drug sensitivity [163]. Similarly, metabolic profiling of breast cancer spheroids identified proline catabolism via Prodh as a critical pathway for survival in 3D environments, with inhibition impairing growth and metastasis [164]. Proteomic studies highlight fundamental differences between 2D and 3D cultures, with spheroids prioritizing tissue-like function over proliferation, showing increased activity in glucose, glycogen, and lipid metabolism [165]. Organoid models of PDAC further revealed metabolic heterogeneity, with glucose transporter 1 (GLUT1), aldolase B (ALDOB), and glucose-6-phosphate dehydrogenase (G6PD)-driven glucose metabolism enhancing chemoresistance [166]. In a microfluidic ductal carcinoma *in situ* model, tumor cells experienced hypoxia-induced metabolic shifts, emphasizing the role of the microenvironment in shaping metabolic adaptations [167]. These findings demonstrate how 3D models capture the metabolic plasticity underlying drug resistance, providing a more physiologically relevant platform for cancer research.

An overview of drug resistance mechanisms and 3D models suitable for studying these mechanisms is presented in Table 1.

## 4 Types of 3D Tumor Models and Their Role in Understanding and Targeting Drug Resistance

### 4.1 Spheroid Models

**Figure 2:**
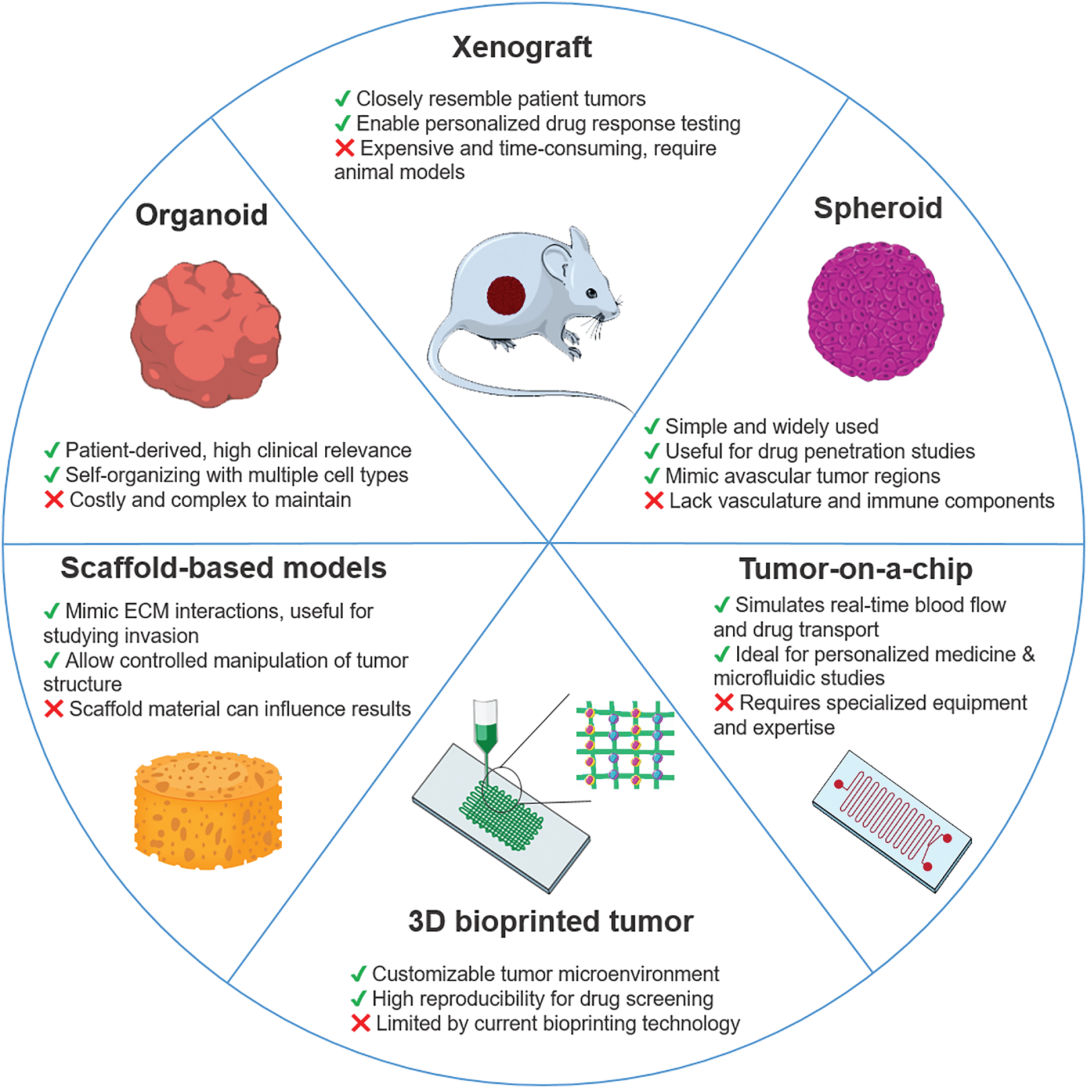
Schematic overview of key 3D cancer models applied in drug resistance studies. Illustrated models include organoids, xenografts, spheroids, tumor-on-a-chip platforms, 3D bioprinted tumors, and scaffold-based systems. Each model is accompanied by its main advantages and limitations in terms of complexity, scalability, physiological relevance, and clinical translatability. This figure was created using images adapted from Servier Medical Art (Servier, smart.servier.com, licensed under a Creative Commons Attribution 3.0 Unported License)

Spheroids are among the most widely used 3D tumor models (Fig. 2). They mimic avascular or poorly vascularized tumors and reproduce cell interactions, nutrient gradients and drug transport limitations. These models are usually made from cancer cell lines or dissociated tumor cells. They are usually over 200 μm in size and consist of three zones: an outer proliferative layer, a middle quiescent zone and an inner necrotic core formed by oxygen and nutrient gradients, which are important for studying the spatial aspects of drug resistance [168]. Techniques for culturing spheroids without scaffolds include spinner flasks, liquid overlay, hanging drops, non-adherent surface culture and aqueous two-phase systems, all of which promote natural cell aggregation [168].

Spheroids are particularly effective for investigating drug resistance mechanisms, as they closely mimic the TME, including drug penetration barriers, intra-tumor gradients, and cellular heterogeneity. Unlike traditional 2D models, spheroids recreate conditions that contribute to therapy failure, making them an essential tool for optimizing treatment strategies and identifying new drug resistance mechanisms.

These advantages have led to a growing body of recent research exploring spheroids as a more accurate model for studying cancer therapies and resistance. Martinez-Bernabe et al. demonstrated that breast and colon cancer spheroids closely mimic *in vivo* conditions and reflect chemotherapy resistance patterns observed in non-responder patients, making them a reliable platform for identifying treatment resistance biomarkers and gaining deeper insights into tumor biology [169]. Using next-generation RNA sequencing, non-malignant (HCEC-1CT) and malignant colon cancer spheroids (HCT116, DLD-1, SW620) were analyzed to identify differentially regulated transcripts that could serve as biomarkers. The expression ratio of transcripts SMAD4-209 and SMAD4-213 was suggested as a biomarker candidate for colon cancer detection, while another study proposed the CD81-215 transcript as a long non-coding RNA of stromal origin with a potential tumor-promoting role in colon cancer [170,171].

Spheroids also mimic the variability of drug exposure in tumors. In ovarian cancer, A2780 spheroids (drug-sensitive and drug-resistant) showed higher resistance to cisplatin and paclitaxel than monolayers due to changes in drug resistance gene expression, extracellular matrix proteins, cell density, and drug diffusion limitations. A moderate increase in cisplatin resistance and a significant increase in paclitaxel resistance were observed in spheroids. These results show how important it is to consider drug penetration and cell dynamics when developing chemotherapies [172]. Additionally, spheroids can model DTP cells that reside in hypoxic or low-drug-exposure areas, which are often associated with relapse and metastasis. These features make spheroids a valuable tool for understanding how tumors develop resistance over time and how specific regions within a tumor contribute to therapy failure. Guillaume et al. demonstrated that growth-induced solid stress significantly altered sensitivity to irinotecan independent of spheroid size, nutrient and oxygen availability, suggesting that mechanical and metabolic stress in tumors contributes to chemotherapy resistance [173].

While most studies use single-cell type spheroids, heterogeneous spheroids with multiple cell types are becoming increasingly popular to better mimic the heterogeneity and physiology of tumors *in vivo*. These models can be used to more accurately study the interactions between tumor cells that are critical for disease progression, response to therapy and drug resistance, and to better mimic tumor-stroma interactions that influence drug sensitivity. Yakavets et al. developed a 3D breast cancer model using heterogeneous spheroids co-cultured with MCF-7 cancer cells and MRC-5 fibroblasts. Compared to monoculture MCF-7 spheroids, co-culture spheroids overexpressed stromal markers like alpha-smooth muscle actin (α-SMA), fibronectin, and collagen, enabling the study of tumor-stroma interactions, especially fibrosis, and testing agents targeting tumor response and anti-fibrotic therapies [174]. Similarly, Lim et al. created heterogeneous spheroids with canine mammary gland cancer cells and macrophages and showed that this method mimics the TME by inducing hypoxia. The study found that hypoxia enhances macrophage-induced chemoresistance, suggesting that inhibition of macrophages is a potential strategy for new cancer therapies [175].

One of the key features of spheroid models is that they can simulate drug penetration through tumor layers, especially in hypoxic regions that contribute to chemoresistance. Many chemotherapies do not reach the tumor core, leaving the inner cells unaffected, which promotes resistance. Spheroids can help analyze drug distribution across tumor regions and develop therapeutic strategies to improve penetration and efficacy. A recent study compared the response to 21 drug combinations in 2D and spheroid cultures of HCT 116, HT-29 and SW-620 cells. The results showed that spheroids were generally more sensitive and exhibited stronger synergistic effects, particularly with mitogen-activated protein kinase kinase (MEK) inhibitor combinations, highlighting the advantage of using spheroids for more accurate drug discovery [176].

Heterogeneous spheroids containing both cancer cells and CAFs are widely used in drug discovery. These models allow a deeper understanding of tumor-stroma interactions and their role in chemoresistance. For example, the therapeutic potential of the small molecule MSI-N1014 was recently investigated with spheroids composed of DLD1 and HCT116 colon cancer cells cultured with fibroblasts transformed into CAFs. MSI-N1014 effectively reduced CAF transformation and increased the sensitivity of cancer cells to 5-FU [177]. Zaghmi et al. have successfully demonstrated that heterogeneous spheroids enable high-throughput screening to investigate the efficacy of anticancer drugs and combination therapies. By co-culturing human neuroblastoma cells with dermal fibroblasts, it was shown that the microsomal prostaglandin E synthase-1 (mPGES-1) inhibitor 934 increases drug binding in tumor cells and thus overcomes the efflux mechanisms responsible for chemoresistance [178].

Spheroids also serve as an effective platform for testing hypoxia-targeting agents and nanoparticle-based drug delivery systems designed to improve drug efficacy in poorly accessible tumor regions. For instance, Fan et al. explored a novel treatment approach by co-targeting CSCs via the ZIP4-HDAC4 axis and non-CSCs using cisplatin for high-grade serous ovarian carcinoma (HGSOC) therapy. They found that ZIP4 induces the sensitization of ovarian cancer cells to histone deacetylase (HDAC) inhibitors. Using a 3D *in vitro* model of HGSOC, they identified HIF-1α and VEGF-A as downstream mediators of HDAC4, which is closely linked to drug resistance and has high clinical relevance. Moreover, they demonstrated that inhibitors of HDAC, HIF-1α, and VEGF-A significantly suppress spheroid formation in ovarian cancer cells PEO4 and PEA2 [179]. A new liposomal system for the hypoxia-activated prodrug tirapazamine (TPZ) was developed and tested on pancreatic tumor spheroids. Using copper complexation (Cu(TPZ)_2_), it improves solubility, uptake, and penetration, overcoming TPZ’s clinical limitations. Additionally, Cu(TPZ)_2_-loaded liposomes sensitize tumor cells to radiation, enhancing therapeutic potential [180].

Nanoparticle-based therapeutics have gained significant attention in recent years, but many promising candidates fail in clinical trials due to limitations of 2D models. In contrast, spheroids offer a more physiologically relevant environment, enabling better predictions of therapeutic effects, assessing nanotoxicity in tumor-like systems. In addition to assessing nanotoxicity, spheroids also serve as valuable models for studying biological barriers. They better reflect cell metabolism, gene expression and signaling pathways, making them useful not only for assessing toxicity but also for evaluating the efficiency of nanoparticle delivery [181].

Spheroids offer advantages such as relevance to tumor biology, cost-effectiveness, reproducibility and compatibility with high-throughput screening and advanced imaging. However, their avascular nature can interfere with cellular processes, affecting the accuracy of drug response, and size variability leads to inconsistent data. In addition, the challenges of evaluating drugs, the difficulty of analyzing single-cell response, and the poor replication of cancer-ECM interactions make standardization difficult, while obtaining cells for molecular analysis remains challenging. While certain spheroid models incorporate stromal components, many lack immune system integration and show variability in cellular composition. Their limited scalability and standardization can also hinder reproducibility and cross-study comparisons.

### 4.2 Organoid Models

Organoids are 3D structures derived from patient tumor samples or stem cells. They self-organize into tissue-like forms while retaining the genetic heterogeneity and complexity of the tumor (Fig. 2). They can contain stromal components and immune cells, making them invaluable for research into cancer biology, drug resistance and personalized medicine. The development of innovative co-culture models, in which cancer organoids are combined with other cells or organoids, can more accurately mimic cell-cell interactions and thus improve the study of tumor behavior and dynamics.

Tumor organoids represent human tumors more accurately than spheroids and preserve genetic diversity, stromal interactions and immune responses [182]. Their ability to retain tumor heterogeneity makes them ideal for assessing response to chemotherapy and studying mechanisms of drug resistance, including EMT, mutation acquisition, and tumor-stroma interactions. Organoids also enable the study of resistance to targeted therapies such as poly (ADP-ribose) polymerase (PARP) or EGFR inhibitors and how tumors adapt to long-term treatment [182]. A recent study by Grunwald et al. used pancreatic ductal carcinoma organoids to investigate the impact of distinct mesenchymal subtypes on tumor progression and drug response [183]. By co-culturing organoids with CAFs from various stromal backgrounds, it was demonstrated that reactive stroma enhances tumor cell proliferation and reduces disease-free survival. Ma et al. used different experimental approaches including HCC PDOs to investigate sorafenib resistance mechanisms in liver cancer [184]. Their study identified glutathione S-transferase A1 (GSTA1) as a key regulator of resistance, suppressing ferroptosis through its peroxidase function. GSTA1 was suggested as a potential biomarker and therapeutic target for overcoming drug resistance in HCC. Recently, Kobayashi et al. established EGFR-mutant lung cancer organoids using HCC827 and H1975 cells, incorporating mesenchymal stem cells and endothelial cells to investigate EGFR-tyrosine kinase inhibitor (TKI) resistance mechanisms in NSCLC [185]. Their findings identified EMT as a key driver of EGFR-TKI resistance. Bevacizumab and miR200c effectively reversed EMT, enhancing drug sensitivity, and were proposed as potential strategies for overcoming chemotherapy resistance in EGFR-mutant NSCLC.

PDOs play a key role in personalized cancer treatment, as they allow clinicians to test how a specific patient’s tumor responds to different drugs. Beyond their growing role in functional precision oncology, PDOs are increasingly recognized as clinically relevant preclinical models with expanding regulatory and translational potential. They are being explored in prospective clinical study settings, where PDOs are tested alongside clinical treatments to inform therapeutic decisions in real time [186]. Furthermore, PDOs contribute to early-phase drug development by supporting high-throughput screening platforms and aiding in the stratification of patient populations for targeted therapies [187]. While standardization for investigational new drug (IND) applications is still underway, initiatives such as the National Institutes of Health (NIH)’s Human BioMolecular Atlas Program (HuBMAP) and the Human Cancer Models Initiative (HCMI) aim to promote regulatory-grade biobanking, data sharing, and protocol harmonization, paving the way for future Food and Drug Administration (FDA) integration [188,189]. As such, PDOs are positioned not only as predictive tools, but also as regulatory-relevant models in next-generation precision oncology pipelines.

PDOs are used in both research and drug testing, with different methods required to produce different cancer organoids. Driehuis et al. provided an overview of the techniques used to produce cancer organoids from patients and the protocols used to test the therapeutic effect [190]. PDOs maintain the genetic profile and heterogeneity of the patient’s original tumor, enabling the identification of therapies that may overcome patient-specific resistance mechanisms. For example, PDOs can predict which drug combinations may be effective against genetic mutations (e.g., EGFR mutations in lung cancer) or resistance pathways (e.g., upregulation of ABC transporters). Ooft et al. demonstrated that PDOs accurately predicted response to irinotecan in 80% of cases of metastatic colorectal cancer, but not to 5-FU or oxaliplatin [186]. In contrast, another study showed that PDOs were good predictors of response to combination treatments of 5-FU, oxaliplatin and irinotecan in colorectal cancer with liver metastases [191]. In addition, primary colorectal cancer PDOs showed the highest sensitivity to 5-FU, with responses varying according to patient characteristics, with cisplatin being more effective in patients younger than 69 years and irinotecan in early-stage disease [192]. Additionally, Wang et al. confirmed that lung cancer PDOs have the ability to predict the effectiveness of treatment options [193].

Recently, Tan et al. screened 419 FDA-approved drugs using liver cancer organoids and identified desloratadine, an antiallergic drug, as a potential anticancer agent [194]. It inhibited tumor growth in organoid, xenograft and cell models by targeting N-myristoyltransferase 1 (NMT1), disrupting visinin-like protein-3 (VILIP3) myristoylation and NFκB/Bcl-2 signaling, suggesting NMT1 as a potential biomarker and therapeutic target in HCC. Scholtes et al. explored preclinical treatments for the AT-rich interactive domain-containing protein 1A (ARID1A)-deficient bladder cancer using PDOs, identifying ARID1A status as a potential biomarker for personalized therapy [195]. They modeled ARID1A loss in normal organoids and found upregulated genes related to DNA repair and cell cycle checkpoints, with prexasertib and YM155 showing potential as treatments. Contreras et al. conducted a drug screening experiment that identified SOS1, SHP2, and broad-spectrum kinase inhibitors, including Nintedanib, as synergistic combination partners for KRASG12C inhibitors in pancreatic ductal adenocarcinoma. Validation in a KRASG12C-mutated PDO model confirmed the efficacy of these combinations [196]. Lee et al. investigated the molecular mechanisms linking myotubularin-related protein 6 (MTMR6) to cisplatin resistance in oral squamous cell carcinoma [197]. Using siRNA knockdown, overexpression strategies, and organoid cultures derived from xenografts and patient tumors, they demonstrated that MTMR6 expression influences cisplatin efficacy, demonstrating its potential as a biomarker for predicting treatment response.

Although organoids mimic important features of tumors, they often cannot fully mimic the vasculature and fluid dynamics that influence drug delivery and resistance *in vivo*. In addition, the immunological microenvironment in organoids may be limited, restricting their ability to model immunological resistance mechanisms such as immune checkpoint evasion. Furthermore, culturing organoids is resource-intensive and requires specialized expertise, which may limit their use in routine drug screening.

### 4.3 Tumor-on-a-Chip Models

Tumor-on-a-chip systems, pioneered by Ingber and colleagues in 2010 with the introduction of the lung-on-a-chip model [198], are microfluidic devices that mimic tumor physiology by integrating multiple cell types in a dynamic, controlled environment. These models simulate aspects of TME such as fluid flow, nutrient gradients and drug exposure and provide more accurate platforms to study tumor biology, drug efficacy, resistance mechanisms and tumor-stroma interactions (Fig. 2). They offer a promising alternative to conventional 2D cultures and animal models for physiologically relevant drug testing [199].

These models excel in simulating drug delivery dynamics, allowing researchers to study how drugs interact with tumor cells in real-time. This is crucial for understanding resistance mechanisms related to drug efflux, tumor heterogeneity, and the formation of vascular barriers, all of which limit drug efficacy. For instance, Komen et al. developed a colorectal cancer-on-a-chip system with a drug-dosing channel for controlled drug delivery. Their study with oxaliplatin showed a dose-dependent inhibition of cell growth, with continuous drug administration proving more effective than a single high-dose treatment [200]. Also, a commercial HepaChip^®®^ system was used to investigate the resistance of PDAC to cisplatin. Their results showed that 3D cultures required significantly higher drug concentrations than 2D cultures to achieve similar levels of cytotoxicity, consistent with patterns found in tumor growth inhibition in mice [201].

To further explore tumor heterogeneity, a microfluidic device was designed to replicate intratumoral heterogeneity in PDAC by culturing cell lines with different genotypes and phenotypes. It was found that resistance to gemcitabine was higher in 3D co-cultures than in 2D monolayers, emphasizing the role of cell-cell interactions in drug resistance [202]. Similarly, Kramer et al. observed that PDAC cells cultured on a chip exhibited a nine-fold higher half-maximal effective concentration for gemcitabine compared to 2D cultures, further supporting the relevance of microfluidic models for resistance studies [203].

Another critical application of tumor-on-a-chip models is studying the role of the blood-brain barrier (BBB) and metastasis in drug resistance. A BBB model examining metastasis of lung, breast and melanoma cancer showed that astrocytes in the BBB modulate permeability and thus influence drug efficacy. Temozolomide was effective both in the presence and absence of a barrier and induced apoptosis in 77% of glioblastoma cells. Cisplatin and carboplatin were only effective in the absence of the BBB, while ifosfamide had no effect [204]. Liu et al. developed a multi-organ microfluidic chip that models brain metastases and reveals resistance to chemotherapy and EGFR inhibitors in brain metastatic PC9 cells. Proteomic analysis linked resistance to oxidative stress and reduced EGFR expression to drug resistance [205–207].

Recent findings highlighted the need for more relevant *in vitro* models. A Eurosarc Phase II trial showed the IGF-1R inhibitor linsitinib ineffective in Ewing sarcoma patients, contradicting monolayer culture and mouse model results. Its cardiotoxicity, linked to IGF-1R signaling in cardiomyocytes, highlights the need for integrated tissue models. A tumor-on-a-chip platform combining Ewing sarcoma and human cardiac muscle replicated clinical outcomes, bridging preclinical and clinical research [208].

Tumors interact closely with their surrounding vasculature, which plays a critical role in drug delivery and resistance. Several tumor-on-a-chip platforms have been developed to study these interactions. One of the strengths of tumor-on-a-chip models is their ability to include stromal cells (e.g., fibroblasts, endothelial cells) and immune cells, which provides a more comprehensive understanding of how TME contributes to drug resistance. Wang et al. constructed a microfluidic chip that simulates the tumor-vascular microenvironment by co-culturing hepatic and breast tumor cell lines with endothelial cells and fibroblasts. Their study revealed that, after the drug treatment, cancer cell viability increased when cultured with endothelial cells and fibroblasts, highlighting the impact of the TME on drug resistance [209]. Nashimoto et al. presented a tumor-on-a-chip platform incorporating a perfusable vascular network, where they tested the effects of paclitaxel on various cancer cell lines. They observed that drug effects in the chip-based system were less pronounced than in static conditions, likely due to improved oxygen and nutrient supply from the vascular network [210]. Lin et al. developed a microfluidic device with a nanoporous membrane between a breast cancer cell chamber and a reservoir to mimic *in vivo* tumor vascularization. The authors compared doxorubicin and paclitaxel effects on static 3D and 2D cultures vs. a microfluidic chip. Both drugs showed increased cytotoxicity with higher concentrations, but effects were stronger in 2D cultures and weaker in the chip, underscoring the value of these models in providing clinically relevant drug response data [211]. Phan et al. developed a vascularized microfluidic platform to evaluate anticancer and anti-angiogenic drug effects on endothelial cells, fibroblasts, and colorectal cancer cells. Their results showed varying drug responses, with some compounds reducing vascular formation, others inhibiting tumor growth, and some more effective in 3D cultures, while others worked better in 2D monolayers [212].

Using tumor-on-a-chip method, researchers can explore how stromal cells promote resistance through ECM remodeling, or how tumor-associated macrophages influence immune evasion and support tumor survival. By simulating immune-related resistance mechanisms, these systems are invaluable for developing therapies that target the TME or enhance the immune response against resistant tumors. For example, resistance to irreversible EGFR-TKIs in NSCLC is closely linked to EMT and driven by the TME. Using a multicellular lung-on-a-chip model, co-culturing human NSCLC cell line HCC827, human fetal lung fibroblasts HFL-1, and human umbilical vein endothelial cells HUVECs, researchers demonstrated that IL-6-mediated fibroblast activation and EMT contribute to osimertinib resistance, which can be partially reversed by IL-6 inhibition with tocilizumab. This microfluidic platform replicates the complex tumor-stroma interactions, offering a valuable tool for studying drug resistance mechanisms and optimizing personalized therapeutic strategies [213]. Furthermore, Wang et al. developed a tumor-microenvironment-on-a-chip device to co-culture macrophages and ovarian adenocarcinoma spheroids in a 3D gel matrix. The macrophages served as carriers of paclitaxel-loaded polymer nanoparticles. The macrophages loaded with the nanoparticles did not cause a reduction in cell viability, but the cytotoxicity on the cancer cells was higher than when administering only the nanoparticles. This model demonstrated the potential for immune cell-mediated drug delivery in tumor-on-a-chip applications [214].

Despite advances, tumor-on-a-chip models face challenges such as standardization, scalability and the difficulty of replicating systemic *in vivo* interactions. Some tumor-on-a-chip platforms are now commercially available, with companies such as Emulate (USA), Mimetas (Netherlands), and CN-Bio (UK) leading the field. For example, Emulate Inc. has commercialized organ-on-a-chip technologies such as Lung-, Liver-, and Gut-on-a-Chip, which are now used by over 150 laboratories worldwide and are supported by the FDA and multiple pharmaceutical companies [198,215,216]. Similarly, Mimetas has developed the OrganoPlate^®^, a high-throughput microfluidic platform that enables continuous perfusion and co-culture of 3D tissues in a 384-well plate format, offering an accessible tool for pharmaceutical research and personalized medicine [217–219]. CN Bio Innovations offers the PhysioMimix^®^ system, a single- and multi-organ microphysiological platform recognized by the FDA and applied in drug metabolism, toxicity testing, and emerging cancer models [220,221]. However, while these platforms provide essential tools for simulating tumor microenvironments and drug responses, highly specialized tumor-on-a-chip systems for studying mechanisms of resistance remain largely prototypical, primarily developed within academic and research settings. The integration of immune components and metastatic processes is still at an early stage. To overcome these limitations, advances in microfluidics, biomaterials and organoid integration are needed. While these models simulate many aspects of drug resistance, they cannot fully capture the evasion of the immune system or the long-term evolution of resistance in solid tumors. Moreover, they may not fully capture the long-term evolution of resistance, particularly in solid tumors that grow over extended periods, as they are typically more challenging to scale for long-term culture. These platforms are technically complex and are not yet widely accessible for routine or large-scale use. In addition, the high cost of fabrication and specialized equipment may limit their cost-effectiveness for broader preclinical screening. Nevertheless, tumor-on-a-chip platforms represent a transformative approach to cancer research, offering more physiologically relevant models for studying tumor behavior, drug efficacy, and resistance mechanisms. By integrating vascular networks, stromal components, and immune cells, these models provide deeper insights into TME interactions. As technology advances, these platforms hold great promise for improving drug development and personalized cancer treatment strategies.

### 4.4 Xenograft Models

Xenograft models represent a critical *in vivo* platform for studying human cancers, as they allow researchers to investigate tumor growth, drug resistance, and interactions within the TME in a whole-organism context. These models involve implanting human tumor cells into immunocompromised mice, enabling the evaluation of therapeutic responses under conditions that mimic aspects of human cancer progression (Fig. 2). The use of xenograft models has provided significant insights into cancer biology, drug efficacy, and mechanisms of resistance [222–224].

Cell line-derived xenografts (CDX) models are established by implanting human cancer cell lines into immunocompromised mice. These models have been widely used for decades to study tumor biology and evaluate novel anticancer drugs. However, one limitation of CDX models is that they do not fully recapitulate the heterogeneity of patient tumors, as they are derived from long-established cell lines with altered genetic and phenotypic properties due to prolonged *in vitro* culture [225,226]. To overcome the limitations of CDX models, PDX models were developed by implanting primary human tumor tissues directly into immunocompromised mice [223,224,227]. These models preserve key characteristics of the original tumor, including genetic heterogeneity and microenvironmental interactions, making them more predictive of clinical responses. PDX models are now widely used in preclinical research for testing targeted therapies, investigating drug resistance, and identifying potential biomarkers for personalized medicine. Their ability to preserve patient-specific tumor heterogeneity and therapeutic response profiles has made them increasingly relevant in clinical translation [224,228]. PDXs are instrumental in preclinical efficacy studies, where they predict clinical responses to investigational drugs and help evaluate therapeutic resistance mechanisms [229]. In co-clinical trials, PDXs are tested alongside patient trials to mirror treatment outcomes and identify predictive biomarkers [230]. These models support IND applications by providing robust, clinically relevant efficacy and pharmacodynamic data [231]. Additionally, PDXs are used for biomarker validation, helping stratify patients for targeted therapies [224], and have emerging applications in personalized medicine, where they guide individualized treatment decisions based on the patient’s own tumor biology.

Xenograft models provide an essential tool for investigating how tumors acquire drug resistance and adapt to therapy over time. By exposing xenografted tumors to various chemotherapeutic agents, researchers can assess mechanisms of resistance, such as enhanced drug efflux, activation of survival pathways, and alterations in the TME. For instance, in breast cancer 1 (BRCA1)-methylated PDX tumors, a resistance mechanism was found where gene fusions repositioned BRCA1 under a different promoter, allowing re-expression despite promoter hypermethylation [232]. This highlights that resistance in BRCA1-deficient tumors can arise from both genetic and epigenetic changes, complicating treatment in BRCA1-mutated breast cancers. Similarly, in the analysis of cetuximab-resistant NSCLC adenocarcinoma PDX models, mesenchymal-epithelial transition factor (MET) overexpression due to gene amplification and activation was identified as a resistance mechanism [233]. MET knockdown sensitized resistant cells to EGF and reduced their anchorage-independent growth and migration, while combination therapy with a MET inhibitor and cetuximab showed additive effects, suggesting a potential therapeutic strategy for MET-driven resistance in lung cancer. Also, in NSCLC, xenograft models were implemented to study BCRP-mediated multidrug resistance. TKI cabozantinib was shown to re-sensitize topotecan-resistant NSCLC xenografts by inhibiting BCRP function and restoring intracellular drug accumulation [234]. PDX models of PDAC were used to evaluate the efficacy of the CHK1 inhibitor AZD7762 in combination with gemcitabine. The study demonstrated that combination therapy suppressed tumor growth more effectively than single-agent treatments and reduced the population of CSCs, which are often implicated in therapy resistance [235]. Similarly, this approach was useful in cancers with poor prognoses, such as metastatic colorectal cancer, where sequential treatment strategies involving PARP inhibitors and oxaliplatin were identified through PDX studies [236]. Furthermore, in colorectal cancer, PDX studies identified HER2 mutations as a cause of resistance to cetuximab, leading to the successful use of HER2-targeted therapies in these cases [236,237]. Similarly, in BRAF-mutant melanoma, PDX models resistant to BRAF and MEK inhibitors exhibited alternative activation of the mammalian target of rapamycin (mTOR) pathway, suggesting that mTOR inhibitors could be a viable treatment option for these patients [238].

PDX models have gained prominence as a translational tool for personalized medicine [224,239]. By creating PDX models from individual patients, researchers can test therapies to identify the most effective treatment for each patient. In uveal melanoma, a genetically stable but highly metastatic cancer, PDX models were successfully developed from 35% of primary tumors and 67% of liver metastases, preserving key genetic and morphological features. Drug testing in these models demonstrated that 31% of high- or intermediate-risk patients could receive treatment recommendations before relapse [228]. In another study, PDX model generated from metastatic, therapy-resistant breast cancer samples successfully recapitulated the disease’s diversity in histologic subtypes and mutational profiles, as confirmed by whole exome sequencing [240]. Integrating genomic and transcriptomic data enabled the identification of oncogenic and actionable pathways in each PDX, facilitating the selection of targeted therapies. PDX-derived short-term *in vitro* cultures retained resistance to paclitaxel chemotherapy, mirroring patient responses. Testing targeted drugs alone or in combination with standard chemotherapy demonstrated the potential of PDXs as a preclinical platform for optimizing personalized treatment strategies in breast cancer [240].

To support preclinical cancer treatment testing, the U.S. and Europe established two multi-center pan-cancer PDX consortia, PDXNet and EurOPDX [229,241]. These efforts also align with FDA priorities for standardizing preclinical models that bridge laboratory and clinical outcomes, highlighting the regulatory and translational relevance of PDXs in oncology drug development. To ensure consistency, Meehan et al. introduced the “PDX models minimal information standard” (PDX-MI standard), covering clinical data, model creation, and quality metrics [242]. In addition to consortium biobanks, various organizations offer PDX platforms with integrated genomic data, such as Mullins et al.’s biobank of 149 diverse patients [243] and Corso et al.’s biobank focusing on microsatellite instability signatures [244]. Conte et al. also launched PDX Finder, a tool for selecting suitable models for specific research [245].

Despite their numerous advantages, xenograft models are not without limitations. One key challenge is the lack of a fully functional immune system in traditional xenografts, which limits their ability to model immune-tumor interactions accurately. Humanized xenograft models help address this issue but are costly and complex to establish. Another limitation is the potential for genetic and epigenetic drift in PDX models over multiple passages, which may affect their ability to faithfully replicate the original tumor’s behavior. They are also expensive, low-throughput, and have long establishment times, limiting rapid translational application. Standardization of PDX generation and characterization is crucial to ensuring reproducibility and reliability in preclinical research. Future advancements in xenograft models may involve more refined humanized models with enhanced immune system functionality, further improving their translational value.

### 4.5 Scaffold-Based 3D Models

Scaffold-based 3D cell cultures have become an important tool in cancer research, especially in the investigation of drug resistance mechanisms. This 3D organization plays a critical role in understanding how cancer cells develop resistance to chemotherapy, targeted therapies and immunotherapies [246]. Scaffold-based 3D models use a supporting matrix or scaffold to culture cells into 3D structures that mimic the architecture and complexity of *in vivo* tumors (Fig. 2) [137,247]. They are useful to study mechanisms of drug resistance such as altered metabolism, ECM remodeling and cell migration, as well as CSCs associated with therapy resistance. These models also simulate *in vivo* conditions and allow the study of drug-ECM interactions and the effects of scaffold properties on resistance mechanisms. By adjusting the matrix composition or stiffness, researchers can test how the ECM environment affects drug resistance and investigate drug combinations or novel delivery systems that target specific resistance pathways.

Scaffolds can be made from either natural or synthetic materials. Natural scaffolds can be made from mammalian ECM biomolecules such as hyaluronic acid, collagen, fibrinogen or Evan basement membrane extracts, but also from natural non-mammalian polymers such as alginate and chitosan [248,249]. The most commonly used synthetic polymers in 3D scaffolds include oly(lactic-co-glycolic acid) (PLGA) [250], polylactic acid (PLA) [251], PLA-polyethylene glycol (PLA-PEG) [252] and poly(ε-caprolactone) (PCL) [253]. While natural scaffolds are often valued for their resemblance to human tissue components, synthetic scaffolds offer distinct advantages, such as better mechanical stability and more adjustable properties. Below, we review recent studies in both types of scaffolds and their contribution to drug resistance research.

A noteworthy example of a natural scaffold-based model is a multicellular 3D heterospheroid tumor system developed in a collagen hydrogel culture. By encapsulating liver cancer heterospheroids cultured together with stromal fibroblasts in a collagen gel, the model mimics the ECM barrier, a key factor influencing drug resistance. Tumors grown in this collagen-based 3D system show greater resistance to anticancer drugs than conventional 2D monolayers and homospheroid cultures [254]. Collagen can be combined with other hydrogels, such as fibrinogen, to further mimic the biomechanical properties of tumor ECM. The model, which included LX2, HepG2, and HUVEC cells in a transmembrane co-culture setup, mimicked the biomechanical properties of fibrotic, cirrhotic and HCC liver tissue. This 3D model exhibited higher resistance to doxorubicin compared to 2D co-cultures, which is very similar to the drug resistance observed in HCC patients [255]. These results suggest that tissue stiffness, ECM composition and stromal architecture are important factors influencing chemoresistance. In a matrigel-based micro-tumor array model, 3D culture conditions were also shown to influence both drug response and tumor cell phenotype. Compared to 2D cultures, tumor spheroids in this system exhibited slower proliferation kinetics and higher reproducibility, which correlated with increased resistance to chemotherapeutics, especially those with weaker potency. This resistance is likely due to the greater heterogeneity of tumor cells and the complex cell-cell and cell-ECM interactions in the 3D microenvironment. In contrast, small-molecule targeted therapies, especially those targeting EGFR, showed higher specificity and sensitivity in EGFR-mutated lung cancer cell lines cultured in the 3D system, suggesting altered receptor expression and signaling compared to 2D models. In addition, drug response results in the 3D model closely matched those of xenografts grown *in vivo*, avoiding 95% of false positives commonly observed in 2D cultures [21]. The importance of ECM for drug resistance was shown by a study in which human decellularized bone scaffolds were used as a model for diffuse large B-cell lymphoma. In this system, lymphoma cells that interacted with both the ECM and mesenchymal stromal cells exhibited increased resistance to doxorubicin-induced apoptosis, particularly in germinal center B-cell lines. These results showed that the ECM and stroma play a critical role in protecting tumor cells from cytotoxic stress [256].

In addition to ECM biomolecules, non-mammalian natural polymers such as alginate and chitosan are also frequently used in scaffold-based 3D cell cultures. Alginate, a polysaccharide derived from brown algae, is a highly biocompatible and easily gel-forming material that provides a hydrated environment ideal for cell encapsulation. It provides a physiologically relevant environment for tumor cell cultures and allows researchers to study the mechanisms of drug resistance more effectively than in conventional 2D models [248]. Several studies have demonstrated the advantages of alginate-based 3D models when it comes to mimicking the interactions within TME, assessing response to chemotherapeutics and detecting resistance-related molecular alterations. In glioblastoma research, alginate scaffolds have been used to create long-term 3D culture models for drug resistance studies. In a study by Dragoj et al., U87 glioblastoma cells were encapsulated in alginate microfibers that maintained cell viability for 28 days. After treatment with temozolomide, genes related to drug resistance (MGMT and ABCB1) were significantly upregulated in 3D cultures compared to 2D models, demonstrating the enhanced resistance profile in alginate-based tumor environments [72]. In the same 3D glioblastoma model, alpha-1 antitrypsin (AAT) expression was significantly upregulated compared to 2D cell cultures, correlating with increased drug-resistance-related gene expression. This suggests that AAT could be a potential biomarker for therapy resistance specifically in 3D models of cancer [257]. To investigate drug resistance in colorectal cancer, Smit et al. developed a 3D alginate model based on a clinostat in which LS180 spheroids encapsulated in sodium alginate were cultured for 20 days. After treatment with paclitaxel, cell growth, viability and glucose consumption decreased, while the expression of P-gp increased—a known resistance mechanism *in vivo* [258].

Chitosan, a biocompatible and biodegradable polysaccharide, has shown promise as a scaffold material for 3D cancer models, especially for drug resistance research. Its porous structure, its ability to support cell adhesion, and its ability to mimic the ECM make it an effective platform for studying tumor-stroma interactions and therapy resistance [249]. A porous 3D chitosan scaffold was used to culture EpCAM(+) and HER2(+) subpopulations of circulating tumor cells from breast cancer, creating a model that closely mimics the microenvironment of aggressive tumors. Compared to 2D cultures, cells in the chitosan-based 3D system exhibited higher expression of pluripotency markers (Nanog, Sox2 and Oct4), enhanced cell-cell and cell-matrix interactions, and increased resistance to doxorubicin [259]. Another recent study by Rezakhani et al. investigated the use of thermosensitive chitosan/β-glycerol phosphate (Ch/β-GP) hydrogel to develop a 3D breast cancer model *in vivo* [260]. This system better mimicked native tumor characteristics and higher expression of cancer stem cell markers (CD44 and CD24) compared to 2D models. Histologic analysis also confirmed that the 3D tumors were more structurally and functionally representative of malignant breast cancer tissue, underscoring the importance of chitosan-based hydrogels for cancer stem cell research and drug resistance studies.

Synthetic polymers have gained a lot of attention in 3D cell culture and tissue engineering due to their adaptability, reproducibility and mechanical stability. Depending on the research application, scaffolds based on synthetic polymers can be customized to support cell growth, mimic the properties of the extracellular matrix, and have adjustable degradation rates, making them versatile tools in biomedical research [261]. Three-dimensional scaffolds based on synthetic polymers such as PLGA, PLA, PLA-PEG and PCL are increasingly used in cancer and tissue engineering research due to their ability to mimic the TME and improve drug resistance. PLGA/PLA-PEG (3P) scaffolds facilitate tumoroid formation and induce EMT, leading to increased chemoresistance [252]. Similarly, electrospun PLA scaffolds, especially with aligned fibers, promote tumor aggressiveness and EMT-related gene expression [262], while PLA nanofiber scaffolds support the growth of triple-negative breast cancer cells and increase signal transducer and activator of transcription 3 (STAT3) activation and CSC markers, making them suitable for short-term 3D culture applications [251].

PLGA scaffolds, which are known for their biocompatibility and biodegradability, have great potential for preclinical drug testing. For example, PLGA/β-TCP scaffolds in perfusion bioreactors simulate mechanically stimulated tumor growth and drug resistance to liposomal doxorubicin, which is very similar to *in vivo* tumor conditions [250]. Similarly, porous PLGA microparticles with interconnected pores have been shown to improve lung cancer cell proliferation and drug resistance, and support scaffold-based drug screening and tissue engineering [263]. Meanwhile, PCL scaffolds have emerged as another promising platform for 3D cancer modeling and regenerative medicine. Multilayered PCL nanofibrous scaffolds successfully mimic the hypoxic microenvironment of tumors, as demonstrated by colon cancer cells exhibiting hypoxia-induced F-actin disorganization and HIF-1α expression, resulting in increased resistance to doxorubicin and ionizing radiation [264]. Similarly, PCL-based scaffolds reinforced with hyaluronic acid create a reproducible glioblastoma model that enables tumor spheroid formation, cell-cell interactions, and ECM-dependent morphological changes [253]. In addition, PCL nanofiber membranes (C-PCL-M and C/DMF-PCL-M) enable the modeling of angiogenesis. They showed that endothelial cell growth varies depending on the composition of the scaffold and the presence of VEGF, with co-culture of cancer cells significantly increasing EC proliferation [265].

Although scaffold-based models offer more *in vivo*-like conditions than conventional 2D cultures, they still have their limitations. A major drawback is that, despite their three-dimensionality, these models cannot fully replicate the complexity of the TME, particularly with regard to vasculature, immune cell interactions, and blood perfusion dynamics, which are critical to understanding drug resistance. While natural-based scaffolds promote cell adhesion and signaling interactions, they also have their pitfalls, such as high compositional variability, limited mechanical strength and potential immunogenicity. Alginate- and chitosan-based scaffolds can suffer from swelling and stability issues that compromise their structural integrity, while limited control over microstructure can affect reproducibility and scalability. Synthetic polymer scaffolds, while adjustable and adaptable for tumor modeling, have difficulty achieving precise architecture, limiting their consistency in preclinical research. In addition, scaffold-based models can be resource-intensive and may require significant optimization for each specific experimental setup. They are also not typically suited for high-throughput screening due to fabrication variability and handling challenges, which may limit their practical utility in early-phase drug testing. Moreover, the absence of integrated immune and vascular components in most setups reduces their ability to capture systemic drug responses or immune-mediated resistance mechanisms. Despite these challenges, scaffold-based models are a powerful tool for studying the mechanical, biochemical and cellular factors that influence drug resistance, even if they cannot fully represent the complexity of a fully functional *in vivo* TME.

### 4.6 3D Bioprinted Tumor Models

Three-dimensional bioprinted tumor models are a powerful tool for studying drug resistance. These models can overcome some of the limitations of conventional scaffold-based systems, such as poor reproducibility and lack of accurate tissue organization. Researchers can manipulate the layers of cells and the surrounding matrix to mimic the tumor’s heterogeneous structure, exploring how these features influence drug penetration and treatment outcomes (Fig. 2). These models are particularly useful for studying resistance mechanisms in specific tumor regions, such as those related to hypoxia and drug efflux, which are key contributors to chemoresistance. Bioprinting enables the creation of patient-specific tumor models that closely mimic the individual characteristics of a patient’s cancer, allowing for personalized and more accurate drug testing [266].

The precision of 3D bioprinting enables the creation of highly individualized tumor models that are ideal for drug delivery studies. These models can replicate the tumor architecture and ECM, which is important for understanding how drugs penetrate the tumor and how resistance develops in different regions. Targeted therapies can be tested in these models to evaluate their efficacy in overcoming spatial barriers to drug penetration, such as the tumor stroma or hypoxic cores.

In glioblastoma models, researchers used bioprinted constructs with glioblastoma stem cells, astrocytes, neural precursor cells, and macrophages within a hyaluronic acid-based hydrogel. These models reflected molecular characteristics seen in patient tumors and supported the study of drug sensitivity, cell communication, and immune cell interactions [267]. In one such model, glioblastoma stem cells printed in hydrogel scaffolds showed the ability to form vessel-like structures and express vascular markers [268], properties known to contribute to resistance by supporting tumor blood supply and adapting to antiangiogenic therapies. Bioprinted glioblastoma models including glioblastoma-associated macrophages have also been used to examine immune-tumor interactions. These models showed that macrophages can support tumor growth and reduce drug sensitivity. Blocking this interaction increased treatment response, supporting the idea that immune cells contribute to resistance in certain tumors [269].

Drug resistance has also been observed in a 3D bioprinted cervical cancer model [270]. HeLa cells embedded in a gelatin-alginate-matrigel matrix underwent EMT when treated with TGF-β, an effect inhibited with agents targeting the EMT signaling pathway, demonstrating the potential for testing anti-metastasis therapies.

Bioprinting has also been used to produce patient-specific HCC models. In a study by Xie et al., tumor cells from HCC patients were mixed with gelatin and alginate to produce bioinks for printing. The resulting 3D structures were shown to have no tumor markers or genetic profiles. These models were stable in long-term cultures and were used for personalized drug screening, demonstrating that 3D bioprinting can be used in personalized medicine [271]. Another HCC study showed that printed HepG2 cells had higher expression of tumor-related and resistance-associated genes than cells in 2D culture. Differences in gene expression were linked to altered responses to chemotherapy [272].

In breast cancer models, different bioprinted tissue structures showed different levels of resistance [273]. Bioprinted solid tumor structures showed higher expression of hypoxia and EMT markers and greater drug resistance compared to ductal structures. These differences were associated with tumor stage and microenvironment. By printing multiple structures together, models mimicking different tumor stages could be created, increasing the relevance for drug response testing.

Intrahepatic cholangiocarcinoma models using patient-derived cells also showed features associated with drug resistance, such as stem cell marker expression and high proliferation [274]. EMT and fibrosis markers were also upregulated. These characteristics may contribute to limited treatment efficacy and highlight the importance of modeling invasive phenotypes *in vitro*. Bioprinting has been used in high-throughput drug screening (HTS) by a system that combines bioprinting with magnetic forces to produce uniform pancreatic cancer organoids in standard HTS formats [275]. This method supported large-scale drug testing and revealed differences in drug responses between 2D and 3D models. The 3D models were more representative of patient-derived tissue and included stromal cells like fibroblasts.

Generally, 3D bioprinted tumor models are a useful tool for studying drug resistance. They allow researchers to control the tumor structure and environment, test patient-specific responses, and study the influence of various factors such as hypoxia, immune cells, and ECM composition. These systems can help improve preclinical testing and support the development of more effective cancer therapies. However, the technology also faces challenges, such as the inability to fully replicate the complex vascular network and immunological microenvironment, both of which are critical to understanding drug resistance *in vivo*. The bioprinting process itself can alter the behavior of cells, including their gene expression, drug response, and viability [276]. Different bioprinting techniques expose cells to distinct forms of stress that may compromise post-printing viability. In nozzle-based methods, such as inkjet and extrusion bioprinting, cells are mainly subjected to shear stress [277,278]. In contrast, light-based methods like laser-assisted bioprinting and stereolithography expose cells to thermal and radiation stress [277,279]. These stresses can disrupt membranes or damage DNA [280], although with optimized conditions, cell viability can still exceed 85–95% depending on the method used [281,282]. The resolution limits can also vary depending on the bioprinting method: while laser-based bioprinting offers high precision, the resolution of other methods such as extrusion printing is usually lower, which can affect the accuracy of microstructural features and limit the modeling of fine tumor structures [283]. In addition, bioprinted tumors, such as spheroids, may not fully capture tumor heterogeneity or other factors such as inflammation that may influence resistance mechanisms. The complexity of bioprinting makes it a resource-intensive technique that is not available in all research facilities and is therefore expensive. Scalability and reproducibility across different printing platforms remain ongoing concerns, limiting its broader adoption in standardized drug screening workflows. Moreover, bioprinted models often need to be complemented by *in vivo* systems or advanced co-culture techniques to capture systemic factors like immune modulation or metastatic behavior. Lack of consensus on standardized bioprinting protocols also complicates cross-study comparisons and broader validation efforts. Despite these limitations, 3D bioprinted cancer models offer a promising platform for drug resistance studies. Table 2 outlines key features, applications, advantages, and limitations of 3D models used in drug resistance research and drug development.

**Table 2:** Comparison of 3D model types in drug resistance research and drug development

3D model type	Key features	Applications	Advantages	Limitations	References
**Spheroids**	Mimic avascular or poorly vascularized tumors Reproduce cell interactions and drug transport limitations Essential for studying therapy failure	Investigating mechanisms of drug resistance Optimizing therapies Identifying resistance pathways High-throughput screening Drug toxicity and nanoparticle delivery evaluation	Closely mimic the tumor microenvironment (TME), including drug penetration barriers, intra-tumor gradients, and cellular heterogeneity	Avascular structure may interfere with drug response Variability in size leads to inconsistent data Difficult single-cell analysis	[169,172,173, 176–181]
**Organoids**	Retain genetic and epigenetic characteristics of primary tumors Model drug resistance and personalized medicine	Drug screening Biomarker identification Combination therapy validation Research into resistance mechanisms and treatment responses	Accurately represent human tumors Preserve genetic diversity and stromal interactions	Limited vasculature Fluid dynamics for drug delivery Immunological microenvironment Resource-intensive culturing	[183–186,190–194]
**Tumor-on-a-chip models**	Suitable for studying drug transport	Studying blood-brain barrier in drug resistance	Simulation of complex interactions in a controlled environment	Challenges in replicating solid tumor complexity	[199,202,204–214]
	Immune cell infiltration Resistance mechanisms associated with intratumoral drug penetration	Stromal cell contribution to resistance ECM and immune influences on tumor behavior Targeted cancer therapies	Involvement of stromal and immune cells Insight into TME’s role in drug resistance	Biological accuracy issues Immune evasion Standardization and scalability problems	
**Xenograft models**	Enable *in vivo* investigation of tumor growth Drug resistance mechanisms Tumor–microenvironment interactions Preserve key tumor characteristics	Preclinical research for targeted therapies Investigating drug resistance Identifying biomarkers for personalized medicine	Preserves tumor heterogeneity (especially patient-derived xenografts (PDXs)) Mimics human tumor progression Predicts clinical responses better than *in vitro* models	Absence of fully functional immune system Reduces immune-tumor interaction modeling Genetic and epigenetic drift in PDXs over passages reduces fidelity	[224,232–239]
**Scaffold-based 3D models**	Mimics *in vivo* tumor microenvironment conditions Investigates tumor cell adaptation to chemotherapy Altered cell architecture, ECM properties, and cell interactions influence resistance	Develop drug delivery systems Targeted therapies Personalized treatment based on tumor characteristics	Closely mimics *in vivo* tumor architecture Investigates altered metabolism, ECM remodeling, cell migration, and CSCs related to resistance	Cannot fully replicate TME complexities Lacks vasculature and immune interactions Natural scaffolds may vary in integrity Synthetic options need optimization	[21,72,248–265]
**3D bioprinted tumor models**	Precisely recreates TME by manipulating cell arrangement and extracellular matrix Reflects individual tumor characteristics	Ideal for drug delivery studies Evaluating targeted therapies Studying immune-tumor interactions Testing anti-metastasis therapies Personalized drug screening	Mimics patient-specific tumor architecture Investigates cellular interactions and drug resistance pathways Predicts therapeutic strategies	Cannot fully replicate complex vascular and immune environments Bioprinting may alter cell behavior, gene expression, and drug response	[267–276]

## 5 Conclusion: Tailoring 3D Model Types to Therapeutic Strategies

In cancer research and drug resistance studies, 3D tumor models have greatly improved our understanding of the complex TME and its role in drug resistance. These models, including spheroids, organoids, xenografts, tumor-on-a-chip, scaffold-based systems and 3D bioprinted models, provide valuable platforms to study the molecular mechanisms behind chemoresistance and evaluate therapeutic strategies with greater accuracy than traditional 2D cultures.

Spheroids and organoids have the advantage of mimicking tumor growth *in vivo*, including cellular heterogeneity and key features of the TME, while xenografts allow the study of human cancer cells in an *in vivo*-like context. Tumor-on-a-chip models, with their ability to mimic dynamic, microfluidic environments, represent a new advance and allow researchers to model the interactions between cancer cells, endothelial cells and immune cells, further enhancing our understanding of the mechanisms of drug resistance. Scaffold-based models, both natural and synthetic, continue to provide versatile platforms that support tumor growth and more accurately mimic tissue structure *in vivo*. Bioprinting technology further refines modeling by enabling precise spatial arrangement of cell types and ECM components, providing unique control over tumor structure for personalized drug testing.

Although these models offer unique advantages, they also present challenges. Spheroids and organoids, while useful, cannot fully replicate the complexity of the TME, particularly with regard to vasculature and immune cell interactions. Xenografts are more complex and costly and require animal models. Tumor-on-a-chip systems offer high adaptability but cannot fully represent the complexity of solid tumors. Scaffold-based models can have issues with material variability and scalability, while 3D bioprinted models are highly accurate but expensive and require advanced technologies.

Ultimately, the selection of a 3D model should be guided by the specific research questions, the type of cancer under investigation and the therapeutic approach to be explored. When researching drug resistance, it is particularly important to match the choice of 3D models to the therapeutic strategy. Spheroids are valuable for studying gradients that drive resistance in solid tumors, while PDOs capture tumor heterogeneity and individual resistance mechanisms. Xenografts validate resistance pathways and therapies *in vivo*. Tumor-on-a-chip systems model dynamic influences of the microenvironment, such as immune and vascular interactions. Scaffold-based models enable the investigation of ECM-mediated resistance, and 3D bioprinting provides spatial control to test drug diffusion and combination strategies. The strategic integration of these platforms increases translational relevance and provides information for resistance-targeted therapies.

A combination of these models could provide the most comprehensive understanding of cancer drug resistance mechanisms and the opportunity to test and develop more effective treatments. The integration of patient-specific models such as bioprinting and personalized cancer organoids promises to revolutionize cancer treatment by predicting individual response, improving preclinical accuracy, reducing clinical trial failures and advancing personalized therapies. Through their advancement, these model systems can accelerate the development of targeted cancer therapies and overcome drug resistance in clinical oncology.

Beyond model selection and integration, several broader challenges and opportunities must be considered to enhance the translational impact of 3D tumor models. One major obstacle in cancer treatment is the limited efficacy of drug delivery, especially in solid tumors with dense extracellular matrix components, abnormal vasculature, and hypoxic regions. These factors often restrict drug penetration and lead to heterogeneous therapeutic responses. Advanced 3D platforms such as perfusable tumor-on-a-chip systems and vascularized bioprinted tumors provide unique opportunities to simulate these barriers and investigate strategies to overcome them, such as nanoparticle-based delivery or localized drug release.

Additionally, the integration of multi-omics technologies, including genomics, transcriptomics, proteomics, and metabolomics, into 3D model workflows offers an unprecedented systems-level view of tumor evolution, resistance mechanisms, and drug responses. Combining these data with patient-specific models like PDOs or bioprinted constructs enables precision oncology efforts that are both mechanistically insightful and clinically actionable. As these models move closer to clinical application, ethical and regulatory considerations are becoming increasingly relevant. Issues such as informed patient consent for tissue-derived models, data privacy, long-term biobanking, intellectual property, and equitable access to advanced model systems must be carefully addressed. Standardization and oversight from regulatory agencies, as well as transparency in data sharing, will be crucial for ensuring the responsible development and implementation of personalized 3D tumor models in both research and clinical contexts.

## Data Availability

Not applicable.
